# Hydrotherapy of Saratoga

**Published:** 1893-02

**Authors:** 


HYDROTHERAPY OF SARATOGA. A Treatise on Natural Mineral Waters. By J. A. IRWIN, M. A., Cambridge, Eng.; M. A., M. D., Dublin University; L. M., College of Physicians, Ire.; Member of Royal College of Surgeons, Eng.; Member of the British Medical Association; Fellow of the London Obstetrical Society, and the New York Academy of Medicine, etc.; formerly House Surgeon Royal Free Hospital, London; Medical Officer Shropeshire and Montgomeryshire Counties Lunatic Asylum, and Physician to the Manchester Southern Hospital for Women and Children. New York: Cassell Publishing Co. 1892.

It is evident that Dr. Irwin is enthusiastic in favor of the medicinal properties of Saratoga waters, and, judging by the comparisons between them and those of the most celebrated European Spaa, not without reason. He thinks that the superior fame of some of the most noted trans-Atlantic waters, such as Carlsbad and others, as curative agents in a certain class of diseases, is, in a great measure, due to the plain food and strict regimen enforced by the doctors in charge, and that, were the same treatment pursued at Saratoga, its waters would yield results equally good, and certainly his reasoning, based, as it is, on the tabulated statements of the analysis of the waters from the most noted springs, both in our country and Europe, seems conclusive.

Valuable hints in regard to the use and abuse of these waters internally administered, are given, but a larger portion of the work is devoted to the science of baking, and to therapeutic applicability, and to dietetics. While deprecating any omnicurative pretensions, denying that they are in any sense specifics, he says, "Judiciously selected and carefully administered they are without exception the safest and most efficient correctives of the morbid constitutional conditions common to most forms of chronic disease, and, hence, may be credited with the widest range of indirect curative power." Sensible dietary directions are given, and general precepts, with a practical division of the waters. The last chapters are devoted to Nosology, showing the range of diseases in which the waters of the different springs are indicated. The book forms a handy volume of 264 pages, small octavo, well printed, indexed, and beautifully bound in cloth.

